# Policosanol Attenuates Statin-Induced Increases in Serum Proprotein Convertase Subtilisin/Kexin Type 9 When Combined with Atorvastatin

**DOI:** 10.1155/2014/926087

**Published:** 2014-11-16

**Authors:** Yuan-Lin Guo, Rui-Xia Xu, Cheng-Gang Zhu, Na-Qiong Wu, Zhi-Ping Cui, Jian-Jun Li

**Affiliations:** ^1^Division of Dyslipidemia, State Key Laboratory of Cardiovascular Disease, Fu Wai Hospital, National Center for Cardiovascular Disease, Chinese Academy of Medical Sciences and Peking Union Medical College, BeiLiShi Road 167, Beijing 100037, China; ^2^Kenli County Hospital, Shandong 257500, China

## Abstract

*Objective*. Statin treatment alone has been demonstrated to significantly increase plasma proprotein convertase subtilisin/kexin type 9 (PCSK9) levels. The effect of policosanol combined with statin on PCSK9 is unknown.* Methods*. Protocol I: 26 patients with atherosclerosis were randomly assigned to receive either atorvastatin 20 mg/d or policosanol 20 mg/d + atorvastatin 20 mg/d for 8 weeks. Protocol II: 15 healthy volunteers were randomly assigned to either policosanol 20 mg/d or a control group for 12 weeks. Serum levels of PCSK9 were determined at day 0 and the end of each protocol.* Results*. Protocol I: atorvastatin 20 mg/d significantly increased serum PCSK9 level by 39.4% (256 ± 84 ng/mL versus 357 ± 101 ng/mL, *P* = 0.002). However, policosanol 20 mg/d + atorvastatin 20 mg/d increased serum PCSK9 level by only 17.4% without statistical significance (264 ± 60 ng/mL versus 310 ± 86 ng/mL, *P* = 0.184). Protocol II: there was a trend toward decreasing serum PCSK9 levels in the policosanol group (289 ± 71 ng/mL versus 235 ± 46 ng/mL, *P* = 0.069).* Conclusion*. Policosanol combined with statin attenuated the statin-induced increase in serum PCSK9 levels. This finding indicates that policosanol might have a modest effect of lowering serum PCSK9 levels.

## 1. Introduction

Proprotein convertase subtilisin/kexin type 9 (PCSK9) is a crucial protein in low-density lipoprotein cholesterol (LDL-C) metabolism by virtue of its pivotal role in the degradation of the LDL receptor [1]. The interactions of statins with PCSK9 are of great interest, not only because statins are the predominant therapeutic agents used to decrease LDL-C and cardiovascular adverse events but also because statin exposure might increase the concentration of serum PCSK9 [2]. Several small studies have found that moderate- to high-dose statins could increase serum PCSK9 levels [3, 4]. In addition, an increased PCSK9 level largely negates further statin-induced increases in the hepatic LDL-C receptor level. This may be a major reason behind statins' “6%” rule in its lipid-lowering effect [5, 6].

How to overcome this side effect of statin is challenging. Policosanol is a natural lipid-modulating drug that slowly and modestly lowers LDL-C, is well tolerated, and has an excellent safety profile. However, little is known about its effects on PCSK9 levels. Our previous study on policosanol showed that it does not increase PCSK9 as much as statins (unpublished data). Atorvastatin is the most widely used statin in many countries, including China [7], and the conventional beginning and maintenance dose for atorvastatin in China is 20 mg/d. We aimed to explore a potentially useful combination lipid-modulating strategy that would not increase PCSK9 levels. In particular, this study was designed to examine the efficacy of a combination of policosanol 20 mg and atorvastatin 20 mg in patients with atherosclerosis in a small, randomized, controlled trial.

## 2. Methods

### 2.1. Study Design and Population

The study protocol was reviewed and approved by the Ethics Committee of Fuwai Heart Hospital, and informed consent was obtained from all patients. Our randomized, controlled, open-label, prospective study consisted of 36 patients with atherosclerosis and 16 healthy volunteers in two protocols. In Protocol I, 36 patients were randomly assigned to receive either atorvastatin 20 mg/d (*n* = 17) or policosanol 20 mg/d + atorvastatin 20 mg/d (*n* = 19) for 8 weeks. In Protocol II, 16 healthy volunteers without any drug treatment within 3 months were randomly assigned to policosanol 20 mg/d or placebo (both *n* = 8) for 12 weeks. Serum lipids levels and serum PCSK9 levels were evaluated at day 0 and at the end of each protocol.

Inclusion criteria of patients for Protocol I were (1) evidence of atherosclerotic lesions on arterial ultrasound, coronary chest tomography, or coronary angiography; (2) no history of statin treatment or use of other drugs known to affect blood lipids within 1 month; (3) ages 18 to 70. Patients with triglycerides (TG) ≥500 mg/dL (5.6 mmol/L), previous acute coronary syndrome within 1 month, serious heart failure or arrhythmia, infectious disease within 1 month, serious liver or renal dysfunction, autoimmune disease, malignant tumor, pregnancy or lactation, or psychiatric disorders were excluded from the study. In addition, patients with one of the following laboratory values above 3 times the upper limit of normal laboratory range: serum glutamic oxaloacetic transaminase, serum glutamic pyruvic transaminase, and creatine phosphokinase above 5 times the upper limit of normal were also excluded.

### 2.2. Laboratory Examinations

Blood samples were obtained from the cubital vein after an overnight fast at day 0, week 8 (Protocol I), or week 12 (Protocol II). All serum samples were stored at –80°C and shipped on dry ice before subsequent analysis. Concentrations of serum total cholesterol (TC), TG, high-density lipoprotein cholesterol (HDL-C), LDL-C, apolipoprotein A1 (ApoA-I), apolipoprotein B (ApoB), lipoprotein (a) (Lp(a)), and free fatty acid (FFA) were measured using an automatic biochemistry analyzer (Hitachi 7150, Tokyo, Japan). TC, TG, HDL-C, LDL-C, and FFA levels were measured using an enzymatic assay. ApoA-I, ApoB, and Lp(a) levels were measured using a turbidimetric immunoassay. Serum PCSK9 concentrations were measured using a high-sensitivity, quantitative sandwich enzyme immunoassay (Quantikine ELISA, R & D Systems Europe Ltd, Uppsala, Sweden) [8]. The threshold for detection was 0.096 ng/mL.

### 2.3. Statistical Analysis

To compare the effects between the treatment groups, we used a simple sample *t*-test. To compare the effects before and after each treatment, we used a paired *t*-test. When the variable did not follow a normal distribution, we used a nonparametric test. For ranked variables, we used a chi square test. Statistical significance was defined as *P* < 0.05. Statistical analysis was performed with SPSS version 17.0 software (Chicago, Illinois, USA).

## 3. Results

Between July 2011 and January 2013, we consecutively screened and enrolled 36 patients with atherosclerosis for Protocol I and 16 healthy volunteers who had taken no drug that affected lipid levels within 3 months for Protocol II from the Division of Dyslipidemia in Fu Wai Heart Hospital. Of the 36 patients, 17 were randomly assigned to receive atorvastatin 20 mg/d group, and 19 were randomly assigned to receive atorvastatin 20 mg/d + policosanol 20 mg/d group for 8 weeks. During the follow-up visit, 5 patients in the atorvastatin group and 5 patients in the atorvastatin + policosanol group were excluded due to the need for coronary artery bypass grafting. The 16 healthy volunteers were randomly assigned to receive policosanol 20 mg/d or placebo (both *n* = 8) for 12 weeks. During the follow-up visit, 1 patient in the policosanol group was excluded due to a possible side effect of an allergy. In the end, a total of 41 subjects (26 for Protocol I and 15 for Protocol II) were analyzed in this study.

### 3.1. Clinical Characteristics of the Patients

The baseline clinical characteristics of the patients enrolled in Protocols I and II are summarized in Tables 1(a) and 1(b). The white blood cell and neutrophil counts were both significantly higher in the atorvastatin group than those in the atorvastatin + policosanol group (*P* = 0.02 and *P* = 0.005, resp.) (Table 1(a)). Subjects were significantly older in the control group than in the policosanol group (*P* = 0.017) (Table 1(b)). As to the other baseline parameters, including inflammatory markers such as the erythrocyte sedimentation rate (ESR) or high-sensitivity C-reactive protein (hsCRP), there was no significant difference between the two groups in either protocol (all *P* > 0.05).

### 3.2. Changes in PCSK9 Levels

**Figure 1:**
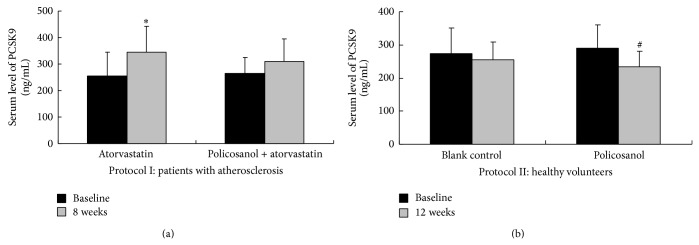
Effect of policosanol on serum levels of PCSK9. (a) In Protocol I, there was no significant difference in baseline serum levels of plasma proprotein convertase subtilisin/kexin type 9 (PCSK9) between the two treatment groups (both *P* > 0.05). Treatment with atorvastatin 20 mg/day for 8 weeks significantly increased serum levels of PCSK9 (*P* = 0.002, shown as “∗”); however, when combined with policosanol 20 mg/day, it caused a trend toward increasing PCSK9, but it was not statistically significant (*P* = 0.184). (b) In Protocol II, there was no significant difference in baseline serum levels of plasma proprotein convertase subtilisin/kexin type 9 (PCSK9) between the two groups (both *P* > 0.05). After treatment with policosanol 20 mg/day for 12 weeks, there was a trend toward decreasing PCSK9 in the policosanol group, although it did not reach statistical significance (*P* = 0.069, shown as “#”).

There was no significant difference in the baseline PCSK9 levels between the two groups in either protocol (all *P* > 0.05). After 8 weeks of treatment in Protocol I, atorvastatin 20 mg/d significantly increased serum PCSK9 levels by 39.4% (from 256 ± 84 ng/mL to 357 ± 101 ng/mL, *P* = 0.002), while policosanol 20 mg/d + atorvastatin 20 mg/d increased serum PCSK9 levels only by 17.4% and this difference did not reach a statistical significance (from 264 ± 60 ng/mL to 310 ± 86 ng/mL, *P* = 0.184). In Protocol II, after 12 weeks of treatment, there was no significant difference in the serum PCSK9 level in the control group (274 ± 77 ng/mL versus 255 ± 54 ng/mL, *P* = 0.212), while there was a trend toward decreasing of serum PCSK9 level in the policosanol group, although it did not reach a statistical significance (289 ± 71 ng/mL versus 235 ± 46 ng/mL, *P* = 0.069) (Table 2 and Figure 1).

### 3.3. Changes in Lipid Profile

**Figure 2:**
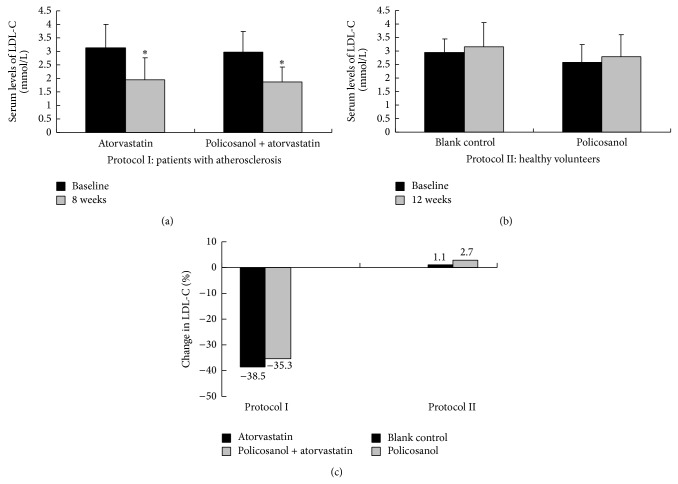
Effect of policosanol on serum lipid levels. (a) In Protocol I, serum low-density lipoprotein cholesterol levels decreased significantly at 8 weeks compared with baseline in both treatment groups (both *P* < 0.01, shown as “∗”). (b) In contrast with Protocol I, serum low-density lipoprotein cholesterol levels did not decrease significantly even at 12 weeks compared with baseline in both treatment groups in Protocol II (both *P* > 0.05). (c) In Protocol I, the extent of the low-density lipoprotein cholesterol decrease between the atorvastatin group and the policosanol + atorvastatin group was not statistically significant (−38.5% versus −35.3%, *P* = 0.692). Similarly, in Protocol II, the change of the low-density lipoprotein cholesterol between the policosanol group and control group was not statistically significant (1.1% versus 2.7%, *P* = 0.769).

**Figure 3:**
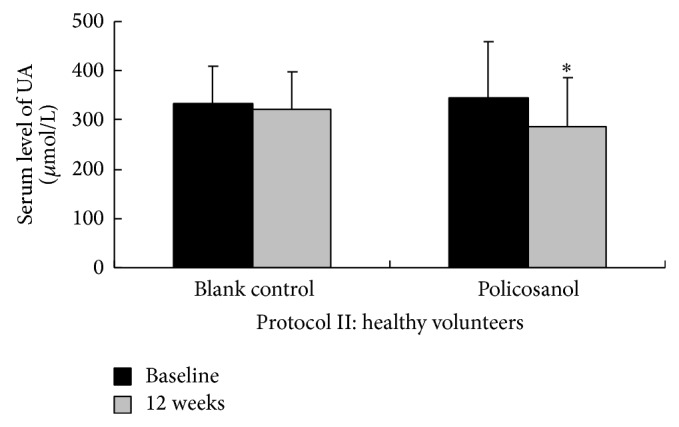
Effect of policosanol on serum levels of uric acid. In Protocol II, we unexpectedly found that policosanol 20 mg/day significantly decreased serum uric acid level at 12 weeks compared with baseline (from 344 ± 115 *μ*mol/L to 288 ± 98 *μ*mol/L, *P* = 0.013, shown as “∗”), while there was no change in the control group (from 323 ± 77 *μ*mol/L to 322 ± 76 *μ*mol/L, *P* = 0.226).

There was no significant difference in the baseline serum level of any lipid parameter in either protocol (all *P* > 0.05) (Table 2). After 8 weeks of treatment in Protocol I, LDL-C decreased 38.5% with atorvastatin 20 mg/d (from 3.18 ± 0.87 mmol/L to 1.97 ± 0.78 mmol/L, *P* < 0.001) and 35.3% with policosanol 20 mg/d + atorvastatin 20 mg/d (from 2.98 ± 0.75 mmol/L to 1.87 ± 0.54 mmol/L, *P* < 0.001). There was no significant difference in the percentage of LDL-C lowering between the two treatment groups (*P* > 0.05). Similar results were found in the changes of other lipid parameters such as TC, TG, HDL-C, Apo AI, ApoB, and FFA. In contrast, after 12 weeks of treatment in Protocol II, there was no significant change in any lipid parameter in either the policosanol group or the control group (all *P* > 0.05) (Table 2 and Figure 2). However, there was an unexpected finding in Protocol II; serum levels of uric acid significantly decreased in the policosanol group (from 344 ± 115 *μ*mol/L to 288 ± 98 *μ*mol/L, *P* = 0.013) compared with control group (from 323 ± 77 *μ*mol/L to 322 ± 76 *μ*mol/L, *P* = 0.226) (Table 2 and Figure 3).

## 4. Discussion

It is well established that statin treatment increases serum PCSK9 levels [9, 10]. Not only does this side effect limit statins' lipid-lowering effect by a double or higher dosage, it also affects its long-term lipid-lowering effect at the same dosage [6, 11]. Conversely, attenuation of PCSK9 function can enhance the hypolipidemic effects of statins [12, 13]. Using a combination of lipid-lowering drugs is a trend in clinical practice, especially for patients who are difficult to reach the LDL-C target level or intolerant to high-dosage statins. Statins are the predominant first-line drug for combining with another lipid-modulating drug. Of course, statins combined with the monoclonal antibody of PCSK9 may be an ideal strategy; however, the monoclonal antibody is not available clinically nowadays, and its long-term safety has yet to be determined [11, 14]. Unfortunately, many other lipid-lowering drugs, such as ezetimibe and fenofibrate, also increase serum PCSK9 levels and increase PCSK9 even more when combined with statins [15–17]. Data on the effects of natural lipid-lowering drugs on PCSK9 are limited. Policosanol is a mixture of higher primary aliphatic alcohols isolated from sugar cane wax, the main component of which is octacosanol. The mixture has been shown to lower cholesterol with excellent safety and tolerance [18].

In our study, in Protocol I, our data confirmed that the conventional dose of atorvastatin 20 mg/day could lower serum LDL-C by 38.5%. Our data also showed that atorvastatin 20 mg/day for 8 weeks increased serum PCSK9 by 39.4%, similar to previously reported levels [3, 4]. However, in our study, we identified that the combination strategy of policosanol + atorvastatin attenuated the extent of the PCSK9 increase that occurs with statins alone. It indicated that policosanol might lower serum PCSK9. Because the study subjects in Protocol I were all patients with atherosclerosis, ethically, we could not use policosanol alone to investigate its effect on PCSK9 in this population. Thus we designed Protocol II of only healthy volunteers as study subjects. Our further data from Protocol II showed a trend toward lowering PCSK9 (*P* = 0.069, compared with baseline). Thus, combining the results of the two protocols, the data indicate that policosanol plus the conventional dose of atorvastatin might be an alternative strategy for attenuating the adverse effects of statins on PCSK9.

However, the other important thing was the lipid-lowering effect using the combination strategy. Data from Protocol I showed a similar lipid-lowering effect between atorvastatin alone group and policosanol plus atorvastatin group. In addition, data from Protocol II in our study showed that policosanol treatment alone for 12 weeks did not significantly affect serum lipid levels. Many studies have demonstrated that policosanol is effective at improving serum lipids, and the lipid benefits were seen in patients with hypercholesterolemia as well as in healthy individuals [19–22]. In recent years, several randomized, controlled trials with small sample sizes reported that policosanol alone or in combination with statins did not affect serum lipid parameters [23–25]. Results from our study also questioned policosanol's lipid modulating effect. Further studies on policosanol treatment for longer time should be carried out to make sure of this point. However, because of the excellent safety and tolerability of policosanol, the search for lipid-lowering compounds based on policosanol is of great importance and interest, especially in children, elderly persons, and other special populations [26–32].

Thus, we believe that policosanol still has a special role in lipid modulation, especially long-term in combination with statins. In addition, regardless of PCSK9 and lipid parameters, we identified an unexpected positive effect of policosanol on uric acid levels [33, 34], which was an extra finding in Protocol II. Another important point in our study is that policosanol, alone or combined with atorvastatin, demonstrated good safety and tolerance in our study. Larger clinical studies on policosanol are needed to confirm and explain our findings.

## 5. Limitations

A small sample size may be a limitation for the present study. This may also add complications of male versus female values and affect our results due to the differences of plasma PCSK9 levels between sexes. Hence, more clinical investigations are needed to confirm our findings.

## 6. Conclusions

In summary, we demonstrated for the first time that policosanol combined with a statin attenuated the statin-induced increase in serum PCSK9 levels; however, the combination treatment did not further lower serum cholesterol levels. Our findings indicate that policosanol might modestly lower serum PCSK9, which might be more important than its lipid-lowering effect for a combination strategy to modulate lipids.

## Figures and Tables

**(a):** 

Variable	Atorvastatin	Policosanol + atorvastatin	*P* value
Number of patients	12	14	
Demographics			
Age (years; mean ± SD)	55.31 ± 6.72	54.73 ± 7.35	>0.05
Men [*n* (%)]	6 (50.0)	11 (78.6)	>0.05
Body mass index (kg/m^2^; mean ± SD)	25.73 ± 2.93	26.26 ± 3.40	>0.05
Clinical profile [*n* (%)]			
Hypertension	7 (58.3)	8 (61.5)	>0.05
Diabetes	4 (33.3)	6 (46.2)	>0.05
Family history of coronary heart disease	1 (8.3)	2 (15.4)	>0.05
Smoker	6 (50.0)	6 (46.2)	>0.05
Laboratory values (mean ± SD)			
White blood cell count (×10^9^/L; mean ± SD)	7.54 ± 1.66	6.15 ± 1.61	0.04
Neutrophils (×10^9^/L; mean ± SD)	4.46 ± 1.05	3.30 ± 1.06	0.01
Erythrocyte sedimentation rate (mm/h; mean ± SD)	12.78 ± 13.09	9.86 ± 10.26	>0.05
high-sensitivity C-reactive protein [mg/L; median (P_25_, P_75_)]	2.86 (1.18, 10.99)	1.27 (0.90, 1.82)	>0.05
Alanine transaminase (U/L; mean ± SD)	28.00 ± 12.10	28.71 ± 15.09	>0.05
Aspartate aminotransferase (U/L; mean ± SD)	19.58 ± 7.86	21.29 ± 10.38	>0.05
Creatinine (*μ*mol/L; mean ± SD)	71.74 ± 14.64	75.20 ± 8.80	>0.05
Blood urea nitrogen (mmol/L; mean ± SD)	5.68 ± 1.36	5.91 ± 1.47	>0.05

**(b):** 

Variable	Control	Policosanol 20 mg	*P* value
Number of patients	8	7	
Demographics			
Age (years; mean ± SD)	45.0 ± 9.9	34.0 ± 3.4	0.017
Men [*n* (%)]	4 (50.0)	4 (57.1)	>0.05
Body mass index (kg/m^2^; mean ± SD)	23.98 ± 1.81	21.76 ± 1.70	>0.05
Clinical profile [*n* (%)]			
Hypertension	1 (12.5)	0 (0)	>0.05
Diabetes	0 (0)	0 (0)	>0.05
Family history of coronary heart disease	0 (0)	1 (14.3)	>0.05
Smoker	2 (25.0)	1 (14.3)	>0.05
Laboratory values			
high-sensitivity C-reactive protein [mg/L; median (P_25_, P_75_)]	1.11 (0.30, 2.66)	0.69 (0.30, 1.43)	>0.05
Fasting blood glucose (mmol/L; mean ± SD)	5.09 ± 0.35	4.84 ± 0.43	>0.05
Alanine transaminase (U/L; mean ± SD)	26.57 ± 17.82	22.14 ± 14.72	>0.05
Aspartate aminotransferase (U/L; mean ± SD)	19.00 ± 6.19	18.57 ± 4.54	>0.05
Creatinine (*μ*mol/L; mean ± SD)	70.06 ± 12.96	71.07 ± 9.91	>0.05
Blood urea nitrogen (mmol/L; mean ± SD)	5.49 ± 1.39	5.25 ± 1.60	>0.05
Uric acid (*μ*mol/L; mean ± SD)	322.6 ± 76.9	344.1 ± 114.8	>0.05

**Table 2:** Effects of policosanol on plasma proprotein convertase subtilisin/kexin type 9, uric acid, and lipid parameters in two protocols.

Variable	I. Patients with atherosclerosis				II. Healthy volunteers			
	Atorvastatin (*n* = 12)		Policosanol + atorvastatin (*n* = 14)		Control (*n* = 8)		Policosanol (*n* = 7)	
	Baseline	8 weeks	Baseline	8 weeks	Baseline	12 weeks	Baseline	12 weeks
PCSK9 (ng/ml; mean ± SD)	256 ± 84	357 ± 101^$^	264 ± 60	310 ± 86	274 ± 77	255 ± 54	289 ± 71	235 ± 46^#^
Uric acid (*μ*mol/L; mean ± SD)	360 ± 70	377 ± 67	332 ± 66	320 ± 89	323 ± 77	322 ± 76	344 ± 115	288 ± 98^*^
Total cholesterol (mmol/L; mean ± SD)	4.91 ± 0.91	3.61 ± 0.85^$^	4.75 ± 0.78	3.35 ± 0.66^$^	5.23 ± 0.54	5.10 ± 0.94	4.94 ± 0.77	4.72 ± 1.00
Triglycerides (mmol/L; mean ± SD)	1.95 ± 0.78	1.46 ± 0.62^$^	1.77 ± 1.20	1.31 ± 0.83^*^	1.59 ± 0.96	1.41 ± 0.76	1.12 ± 0.47	0.94 ± 0.15
HDL-C (mmol/L; mean ± SD)	1.04 ± 0.23	0.98 ± 0.26	1.16 ± 0.27	1.12 ± 0.21	1.31 ± 0.42	1.35 ± 0.40	1.61 ± 0.46	1.54 ± 0.40
LDL-C (mmol/L; mean ± SD)	3.12 ± 0.89	1.97 ± 0.78^$^	2.98 ± 0.75	1.87 ± 0.54^$^	2.94 ± 0.50	3.16 ± 0.89	2.57 ± 0.67	2.78 ± 0.83
Apoprotein AI (g/L; mean ± SD)	1.61 ± 0.31	1.35 ± 0.34	1.46 ± 0.27	1.31 ± 0.34	1.71 ± 0.36	1.67 ± 0.32	1.87 ± 0.40	1.71 ± 0.26
Apoprotein B (g/L; mean ± SD)	1.29 ± 0.38	0.75 ± 0.26^$^	1.08 ± 0.23	0.65 ± 0.22^$^	0.83 ± 0.12	0.88 ± 0.20	0.70 ± 0.11	0.76 ± 0.15
Free fatty acid (mmol/L; mean ± SD)	0.50 ± 0.12	0.62 ± 0.20	0.36 ± 0.13	0.32 ± 0.25	0.39 ± 0.14	0.38 ± 0.13	0.49 ± 0.42	0.45 ± 0.16

^*^ *P* < 0.05 versus baseline; ^$^ *P* < 0.01 versus baseline; ^#^ *P* = 0.069 versus baseline.

PCSK9, proprotein convertase subtilisin/kexin type 9; HDL-C, high-density lipoprotein cholesterol; LDL-C, low-density lipoprotein cholesterol.

## References

[1] Mousavi S. A., Berge K. E., Leren T. P. (2009). The unique role of proprotein convertase subtilisin/kexin 9 in cholesterol homeostasis. *Journal of Internal Medicine*.

[2] Khera A. (2012). Statins, plasma proprotein convertase subtilisin/kexin type 9 concentrations, and LDL lowering. *Clinical Chemistry*.

[3] Careskey H. E., Davis R. A., Alborn W. E., Troutt J. S., Cao G., Konrad R. J. (2008). Atorvastatin increases human serum levels of proprotein convertase subtilisin/kexin type 9. *Journal of Lipid Research*.

[4] Welder G., Zineh I., Pacanowski M. A., Troutt J. S., Cao G., Konrad R. J. (2010). High-dose atorvastatin causes a rapid sustained increase in human serum PCSK9 and disrupts its correlation with LDL cholesterol. *The Journal of Lipid Research*.

[5] Dong B., Wu M., Li H., Kraemer F. B., Adeli K., Seidah N. G., Park S. W., Liu J. (2010). Strong induction of PCSK9 gene expression through HNF1*α* and SREBP2: mechanism for the resistance to LDL-cholesterol lowering effect of statins in dyslipidemic hamsters. *Journal of Lipid Research*.

[6] Konrad R. J., Troutt J. S., Cao G. (2011). Effects of currently prescribed LDL-C-lowering drugs on PCSK9 and implications for the next generation of LDL-C-lowering agents. *Lipids in Health and Disease*.

[7] Guo Y.-L., Liu J., Li J.-J., Zhu C.-G., Qing P., Jia Y.-J., Wu N.-Q., Nie S.-P., Li Z.-C., Zeng H.-S., Yang P. (2011). A multi-center survey of achieving recommended lipid goals in Chinese patients with coronary artery disease in real world cardiovascular practice. *International Journal of Cardiology*.

[8] Dubuc G., Tremblay M., Paré G., Jacques H., Hamelin J., Benjannet S., Boulet L., Genest J., Bernier L., Seidah N. G., Davignon J. (2010). A new method for measurement of total plasma PCSK9: clinical applications. *Journal of Lipid Research*.

[9] Awan Z., Seidah N. G., MacFadyen J. G., Benjannet S., Chasman D. I., Ridker P. M., Genest J. (2012). Rosuvastatin, proprotein convertase subtilisin/kexin type 9 concentrations, and LDL cholesterol response: the JUPITER trial. *Clinical Chemistry*.

[10] Huijgen R., Boekholdt S. M., Arsenault B. J., Bao W., Davaine J.-M., Tabet F., Petrides F., Rye K.-A., Demicco D. A., Barter P. J., Kastelein J. J. P., Lambert G. (2012). Plasma PCSK9 levels and clinical outcomes in the TNT (Treating to New Targets) trial: a nested case-control study. *Journal of the American College of Cardiology*.

[11] Cariou B., Le May C., Costet P. (2011). Clinical aspects of PCSK9. *Atherosclerosis*.

[12] Rashid S., Curtis D. E., Garuti R., Anderson N. H., Bashmakov Y., Ho Y. K., Hammer R. E., Moon Y.-A., Horton J. D. (2005). Decreased plasma cholesterol and hypersensitivity to statins in mice lacking Pcsk9. *Proceedings of the National Academy of Sciences of the United States of America*.

[13] Stein E. A., Gipe D., Bergeron J. (2012). Effect of a monoclonal antibody to PCSK9, REGN727/SAR236553, to reduce low-density lipoprotein cholesterol in patients with heterozygous familial hypercholesterolaemia on stable statin dose with or without ezetimibe therapy: a phase 2 randomised controlled trial. *The Lancet*.

[14] Vogel R. A. (2012). PCSK9 inhibition: the next statin?. *Journal of the American College of Cardiology*.

[15] Costet P., Hoffmann M. M., Cariou B., Delasalle B. G., Konrad T., Winkler K. (2010). Plasma PCSK9 is increased by Fenofibrate and Atorvastatin in a non-additive fashion in diabetic patients. *Atherosclerosis*.

[16] Mayne J., Dewpura T., Raymond A. (2008). Plasma PCSK9 levels are significantly modified by statins and fibrates in humans. *Lipids in Health and Disease*.

[17] Davignon J., Dubuc G. (2009). Statins and ezetimibe modulate plasma proprotein convertase subtilisin kexin-9 (PCSK9) levels.. *Transactions of the American Clinical and Climatological Association*.

[18] Janikula M. (2002). Policosanol: a new treatment for cardiovascular disease?. *Alternative Medicine Review*.

[19] Gouni-Berthold I., Berthold H. K. (2002). Policosanol: clinical pharmacology and therapeutic significance of a new lipid-lowering agent. *American Heart Journal*.

[20] Castaño G., Mas R., Fernández L., Illnait J., Mesa M., Alvarez E., Lezcay M. (2003). Comparison of the efficacy and tolerability of policosanol with atorvastatin in elderly patients with type II hypercholesterolaemia. *Drugs and Aging*.

[21] Reiner Ž., Tedeschi-Reiner E., Romić Ž. (2005). Effects of rice policosanol on serum lipoproteins, homocysteine, fibrinogen and C-reactive protein in hypercholesterolaemic patients. *Clinical Drug Investigation*.

[22] Chen J. T., Wesley R., Shamburek R. D., Pucino F., Csako G. (2005). Meta-analysis of natural therapies for hyperlipidemia: plant sterols and stanols versus policosanol. *Pharmacotherapy*.

[23] Dulin M. F., Hatcher L. F., Sasser H. C., Barringer T. A. (2006). Policosanol is ineffective in the treatment of hypercholesterolemia: a randomized controlled trial. *The American Journal of Clinical Nutrition*.

[24] Berthold H. K., Unverdorben S., Degenhardt R., Bulitta M., Gouni-Berthold I. (2006). Effect of policosanol on lipid levels among patients with hypercholesterolemia or combined hyperlipidemia: a randomized controlled trial. *The Journal of the American Medical Association*.

[25] Backes J. M., Gibson C. A., Ruisinger J. F., Moriarty P. M. (2011). Modified-policosanol does not reduce plasma lipoproteins in hyperlipidemic patients when used alone or in combination with statin therapy. *Lipids*.

[26] Martino F., Puddu P. E., Pannarale G. (2013). Low dose chromium-polynicotinate or policosanol is effective in hypercholesterolemic children only in combination with glucomannan. *Atherosclerosis*.

[27] Marazzi G., Cacciotti L., Pelliccia F., Iaia L., Volterrani M., Caminiti G., Sposato B., Massaro R., Grieco F., Rosano G. (2011). Long-term effects of nutraceuticals (berberine, red yeast rice, policosanol) in elderly hypercholesterolemic patients. *Advances in Therapy*.

[28] Affuso F., Ruvolo A., Micillo F., Saccà L., Fazio S. (2010). Effects of a nutraceutical combination (berberine, red yeast rice and policosanols) on lipid levels and endothelial function randomized, double-blind, placebo-controlled study. *Nutrition, Metabolism and Cardiovascular Diseases*.

[29] Cicero A. F. G., Rovati L. C., Setnikar I. (2007). Eulipidemic effects of berberine administered alone or in combination with other natural cholesterol-lowering agents: a single-blind clinical investigation. *Arzneimittel-Forschung/Drug Research*.

[30] McCarty M. F. (2005). An ezetimibe-policosanol combination has the potential to be an OTC agent that could dramatically lower LDL cholesterol without side effects. *Medical Hypotheses*.

[31] (2006). New policosanol product combines natural cholesterol lowering with omega-3 fatty acids to lower CV risk. *Cardiovascular Journal of South Africa*.

[32] Fontani G., Lodi L., Migliorini S., Corradeschi F. (2009). Effect of omega-3 and policosanol supplementation on attention and reactivity in athletes. *Journal of the American College of Nutrition*.

[33] Torres O., Agramonte A. J., Illnait J., Ferreiro R. M., Fernandez L., Fernandez J. C. (1995). Treatment of hypercholesterolemia in NIDDM with policosanol. *Diabetes Care*.

[34] Zardoya R., Tula L., Castaño G., Más R., Illnait J., Fernández J. C., Díaz E., Fernández L. (1996). Effects of policosanol on hypercholesterolemic patients with abnormal serum biochemical indicators of hepatic function. *Current Therapeutic Research—Clinical and Experimental*.

